# First Distrtct Dental Society of the State of New York

**Published:** 1871-12

**Authors:** S. G. Perry


### FIRST DISTRICT DENTAL SOCIETY OF THE STATE OF NEW YORK.

This Society met October 3d, 1871.

Dr. Atkinson read the following paper on "Smooth Points:"

### SMOOTH POINTS.

However grandiloquently novices, freshmen, and sophomores may plume themselves upon their ability to understand, take sides, and defend by proof the aspect of all questions that arise in nature, including the whole range of physics, psychics, and the processes thereto belonging, still there is, outside of the realm of numbers, a persistent remainder of unproved correlation of the elements of propositions, in excess or deficiency, that prevents full satisfaction to the mind of him who desires to *know*.

Those who attempt to formulate the mutations of force and form in nutrient or mechanical function in mathematical figures without loss or remainder, may look for disappointment in the effort. The laws of progress have indelibly written *change* on all bodies and processes in Nature, whether they consist in separate or conjoined domains of science and philosophy, of principles and practice, pertaining to the evolutions of the physical, mental, moral, or spiritual unfoldment of pre-ordained plan and process, in general and in particular, in persons and professions.

The concepts of principle and the performance of process, at each stage of evolution of plan, purpose, and destiny, are necessarily limited to their natural range, and it is impossible to separate or ignore the serial order, without arresting or destroying the career of progress.

Professions, like individuals, have an origin, growth, culmination, and end. The aspects through which every body or process passes in its career are regular in succession of stage of degree and development of the plan or type, without which they cannot exist as body or process.

The different periods of dentistry are calculable from the character of operations and the instruments used. The performances for the preservation of decayed teeth now executed, in the hands of the best operators was an impossibility, but a short time since, in any hands. "Smooth Points" is very indefinite and evasive, as an adequate description of the instruments used to insert gold in cavities in teeth. Almost all the phrases employed to describe instruments and processes and methods of use are liable to fail to convey the concept of the speaker or writer; so that efficient teaching involves the presence of the thing described, whether that be an implement or a performance. Hence didactic teaching should be superseded by the exhibition of means and methods in actual service, in demonstrating their value and power in open clinic to all.

As all advice is for those ready to adopt it, the question of the advisability of advising is one of the degree of the evolution of the states of those to whom the advice is proffered; hence those only who are not complete in conception and execution of the work of filling teeth with gold may hold themselves as legitimately addressed upon this complex unity or unitary complexity.

Many who are not satisfied with their attainments are, nevertheless, very "conservative," as they say, and make objections to leaving the old unsatisfactory methods, for the sake of "consistency" and "reliability," in not transcending the limits of their former unsatisfactory performances.

What is it that is conserved in such cases other than error and failure to accomplish the good already conceived? The dental profession, like all other offshoots of the necessities of mankind, began at its lowest estate, and can only rise to its highest by untiring effort.

Those who tarry in what may be called the barely *physical* stage of professional development, will persistently call it a mere "finger craft," while those who rise to the *mental* stage will perceive the necessity of understanding the principles that underlie the mere how to do it. And these will enable us to recognize the *moral* stage of professional development, which will naturally lead us into the domain of the spiritual and æsthetic stage of professional evolution.

Thus the truly æsthetic stage includes all the preceding, and involves not only the how, but the why and when of all the performances included in the understanding, preparation, and execution of the redemptive work. Blessed is he whom the *truth* doth not offend. If plain expression of truth convicts us of deficiencies, and we are not willing to learn to overcome them, our stage of growth is low, and our works in accordance with the degradation of standard adopted.

Filling teeth with "smooth"-ended instruments was practiced in earliest dawn of the art, and had any one using them emerged out of the physical stage, there would have been opened a way to mental, moral, and æsthetic stages long since.

Let us not find fault and kick against progress, but humbly acknowledge the right and use of each stage, and thus accelerate the impetus that shall bring us all to use smooth points and heavy gold, and do perfect work.

Dr. J. S. Latimer asked, why not start a filling with serrated points and finish with smooth ones?

Dr. Atkinson replied there was no necessity, as a filling could be started by using four or five ply of 60 to 120 gold, holding it in place until anchored, and then finishing throughout with smooth points. The idea of using gold so heavy first came to him by melting a pellet into the form of wire while annealing it. He put it in the center of the filling, and found that it went down easily under the instrument.

In the old way we didn't make solid fillings—there were many crevices—the gold was *interdigitated*, not welded. Welding is the annihilation of space. Flux is used only to make a clean surface, and it is a mistake to suppose that heat is necessary at the moment of welding. Didn't care to have the center of fillings solid.

If the patient and operator didn't harmonize the patient ought to be dismissed till another time; or if there was permanent mental and spiritual clashing, given into the hands of another man. One should never operate unless sure of a good result, and unless the patient and operator work together that could not be attained. If men would learn to do difficult work they must see it done. Believed there were more doing good work now than ever before. Had prayed that he might be the *best* dentist on the planet. Didn't care if he was criticised; for he said these things for the reason that he had seen the time when he would have licked any one's boots to have been told the same. He had now transcended what were then his best ideals.

Dr. Fitch said he had no doubt every honest dentist, coupled with intelligence, had made the same prayer during his whole life. His first idea in reference to smooth points and heavy gold came from heating pellets in the lamp and having them fall from the pliers upon pieces of gold, to which they adhered. If they would do this, why use serrations? and this led him to experiment in that way. Had better success with heavy than with light gold. Get better margins with smooth points. Didn't use burnished points, but those flat on the surface and filed nearly smooth. Had found that he could not pack gold in some mouths without the rubber dam, but did not always use it, nor did he always use the mallet.

Dr. Taft, being present, said smooth points were just coming in vogue; was not aware that any article, except one, had been written claiming their superiority. Only a few men here and there believed that gold could be welded with them. He had been surprised during the past year at the manner in which he had found gold would weld under smooth instruments. He had been convinced on this subject. Experiment demonstrates it.

Had often been obliged to change opinions, and might have to do so again. Was ready to do so when facts require it. Preferred smooth points to serrations, chiefly because of their thorough condensation of the gold about the margins of the cavity. Gold laid against the borders of cavities, or against the enamel, is often perforated by serrated points, and there is danger of injury thereby. In such cases the microscope will show broken or marred borders that ultimately disintegrate. Considered this often the cause of the discoloration that eventually appears about some fillings. This can be obviated by the use of smooth points. His ordinary method was to fill the body of the cavity with No. 20 to 30, but when near the margins uses No. 60, letting it overlap, and forging up against the border with smooth instruments. Also adopts this method in building on or overlapping the edge of cavities that have been cut or filed, in such a way as to protect the cut surface. Uses in this way from eight to ten ply of No. 60.

Gold could be built up with smooth points; had tried it, and had succeeded beyond his expectations. Found it hard to tear gold away from a smooth surface, so perfectly could it be welded. It would tear in two before it would come off.

He would take exception to one point in the paper just read; that is the prominence given to clinical teaching over didactic. A few men naturally learn all they know through their eyes, but he thought it better to learn through the intellect as well. But few could learn to do difficult work by simply seeing it done. Operations in the mouth were not repetitions of the same thing. There were fixed principles underlying them, and these principles must be learned first. The mind first conceives and then the hand works out the conception, and until the directing power is properly educated and balanced the hand is powerless. There are some who can best learn by demonstration, but to be able to meet *all* conditions, principles must be first learned.

Dr. Latimer asked if he understood Dr. Taft to say he let the gold overlap the edge of all cavities.

Dr. Taft—Yes, so far as the teeth have been cut. Considered that it should be a principle in filling to always restore the original shape of the tooth. Some circumstances forbid this, but should do so where it could be done. Would not transcend it unless in exceptional cases. Would not let the gold overlap the enamel in crown-surface cavities, unless where it was necessary to secure a better occlusion. Would simply say that he would protect every exposed portion, and this would be done by restoring the original shape of the tooth. And this can better be done since the advent of heavy gold.

In reply to a question, said he used Guillois' cement in the bottom of large cavities, if the teeth were frail or the patient unsuited to a lengthy, tiresome operation, cutting away portions of the cement and filling the remainder of the cavity with gold. Would endeavor to use discretion, putting the cement in teeth not much used, while for a strong man, whose teeth close with much force, would use more gold.

Dr. Thomas, of Madrid, Spain, said what he had heard came to him as a new light. It was food for a starving soul. It made him feel that he was a long way behind the times. Had found, in Spain, that chalky decay predominates. Thought the pulps of teeth larger in tropical countries. Had found them easily, and often exposed. Said the water of Madrid was hard, and considered that there were more teeth lost from salivary calculus than by decay.

Dr. Latimer, in reference to the prayers of Drs. Atkinson and Fitch, said that he had prayed that he might be a *good* dentist, but he had never prayed that he might be the best. If each of us should pray to the Good Father that we might be the *best*, what could we expect He would do about it? Said he had rather pray that he might serve his patients well and faithfully.

Dr. Francis thought dentists the greatest set of innovators on earth. They were always after something new, and when anything new was announced there was always some one ready to claim a previous discovery of it. Formerly soft foil was used, then adhesive, and now some are advocating soft foil again. We had thin gold and now thick, and some have become sick of it and have gone back to thin. And the same changes have occurred in the kinds of points used. Was a conservative, and believed truth would be found between the two extremes. Formerly he used such serrations as were found at the depots, but now uses much finer ones. Prefers them as fine as could be made. Was not in favor of heavy foil. Wanted a foil that could be perfectly adapted to the walls of the cavity. Spoke of a tin filling that had remained perfect for sixty years. How often had we all seen tin fillings that had remained twenty or thirty years? Believed the reason to be the perfect adaptation attained by the use of this metal. Didn't believe thick gold could be perfectly adapted to the walls. As he had said before, if he was required to fill an egg shell with paper he should use tissue, not pasteboard. Didn't believe as much adhesive gold was used now as formerly. Uses soft foil against the walls and adhesive in the center. Had seen fillings, from the hands of our best operators, where the gold came out solid, like a bullet, and believed it due to adhesive gold. Said it had been proven that the heavier the gold the less could be packed into a cavity drilled in a steel plate.

Dr. Fitch said we should look at this matter in a practical way. The general way to preserve a tooth from decay was to exclude moisture, and his aim was to pack gold in such a way as to attain that end. Thought any one who could not use adhesive gold against the walls in that way ought not to use it. He didn't care for the demonstration if he didn't understand the principle.

Dr. Taft said a knowledge of conditions—a knowledge of the management of conditions, physiological and pathological—was necessary in the treatment of diseases of the mouth. All failures were *not* due to heavy gold. We must take into account the susceptibility of teeth to decay, the lack of care on the part of the patient—in fact, many things that conspire to make even the most careful operations failures. Our manipulative ability is far in advance of our knowledge of principles. It is not safe to do for one what it may be well to do for another. We must learn to recognize these differences of conditions.

S. G. PERRY, *Sec'y.*

